# The Onset of Antinuclear Antibodies (ANAs) as a Potential Risk Factor for Mortality and Morbidity in COVID-19 Patients: A Single-Center Retrospective Study

**DOI:** 10.3390/biomedicines12061306

**Published:** 2024-06-13

**Authors:** Giuseppe Stefano Netti, Piera Soccio, Valeria Catalano, Federica De Luca, Javeria Khalid, Valentina Camporeale, Giorgia Moriondo, Massimo Papale, Giulia Scioscia, Gaetano Corso, Maria Pia Foschino, Sergio Lo Caputo, Donato Lacedonia, Elena Ranieri

**Affiliations:** 1 Unit of Clinical Pathology, Advanced Research Center on Kidney Aging (A.R.K.A.), Department of Medical and Surgical Sciences, University of Foggia, 71122 Foggia, Italy; valeria.catalano@unifg.it (V.C.); federica.deluca@unifg.it (F.D.L.); javeria.khalid@unifg.it (J.K.); valentina.camporeale@unifg.it (V.C.); elena.ranieri@unifg.it (E.R.); 2 Unit of Clinical Pathology, Department of Laboratory Diagnostics, University Hospital “Policlinico Riuniti”, 71122 Foggia, Italy; massimo.papale@unifg.it (M.P.); gaetano.corso@unifg.it (G.C.); 3 Unit of Respiratory Medicine, Department of Medical and Surgical Sciences, University of Foggia, 71122 Foggia, Italy; piera.soccio@unifg.it (P.S.); giorgia.moriondo@unifg.it (G.M.); giulia.scioscia@unifg.it (G.S.); mariapia.foschino@unifg.it (M.P.F.); donato.lacedonia@unifg.it (D.L.); 4 Clinical Biochemistry, Department of Clinical and Experimental Medicine, University of Foggia, 71122 Foggia, Italy; 5 Unit of Infectious Diseases, Department of Medical and Surgical Sciences, University of Foggia, 71122 Foggia, Italy; sergio.locaputo@unifg.it

**Keywords:** COVID-19, SARS-CoV-2, ANA, antinuclear antibodies, HALP score

## Abstract

The immune system’s amplified response to SARS-CoV-2 may lead to the production of autoantibodies, but their specific impact on disease severity and outcome remains unclear. This study aims to assess if hospitalized COVID-19 patients face a worse prognosis based on ANA presence, even without autoimmune diseases. We performed a retrospective, single-center, observational cohort study, enrolling 638 COVID-19 patients hospitalized from April 2020 to March 2021 at Hospital “Policlinico Riuniti” of Foggia (Italy). COVID-19 patients with a positive ANA test exhibited a significantly lower 30-day survival rate (64.4% vs. 83.0%) and a higher likelihood of severe respiratory complications during hospitalization than those with negative ANA screening (35.4% vs. 17.0%) (*p* < 0.001). The association between poor prognosis and ANA status was identified by calculating the HALP score (Hemoglobin-Albumin-Lymphocyte-Platelet), which was lower in COVID-19 patients with a positive ANA test compared to ANA-negative patients (108.1 ± 7.4 vs. 218.6 ± 11.2 AU; *p* < 0.011). In detail, COVID-19 patients with a low HALP showed a lower 30-day survival rate (99.1% vs. 83.6% vs. 55.2% for high, medium, and low HALP, respectively; *p* < 0.001) and a higher incidence of adverse respiratory events compared to those with high and medium HALP (13.1% vs. 35.2% vs. 64.6% for high, medium, and low HALP, respectively; *p* < 0.001). In summary, ANA positivity in COVID-19 patients appears to be linked to a more aggressive disease phenotype with a reduced survival rate. Furthermore, we propose that the HALP score could serve as a valuable parameter to assess prognosis for COVID-19 patients.

## 1. Introduction

Severe acute respiratory syndrome virus coronavirus 2 (SARS-CoV-2) infection can cause irreparable damage to the human body, affecting the functioning of the immune system and triggering, in the medium- and long-term, the development of autoimmune diseases [1,2,3,4].

The key role that the immune system plays in SARS-CoV-2 disease has been evident since the early months of the pandemic. In severe COVID-19 cases, autoantibodies were found to mediate a destructive autoimmune response, as demonstrated by several studies [5,6,7].

An increase in the frequency of anti-phospholipid antibodies (APLs) was observed in COVID-19 patients, showing that these autoantibodies are related to a hyper-inflammatory state with extremely high levels of ferritin, C-reactive protein, IL-6, and pulmonary thromboembolism [8]. Autoantibodies for annexin-A2, the pulmonary protective protein, were found to be linked to patient mortality in COVID-19 [9]. Moreover, the severity of the disease among COVID-19 patients was explained by the identification of autoantibodies against both type I interferons and cytokines [10,11].

Recent studies found that ANAs could be a potential prognostic factor [12] and that ANAs may reflect immune dysregulation caused by the virus [13].

Taking into account these considerations, with approximately 20% of COVID-19 patients at our center testing positive for antinuclear antibodies (ANAs), our primary aim was to investigate the clinical implications of this phenomenon through a retrospective, observational clinical study. In detail, the main goal of the study was to evaluate how the onset of ANAs in patients hospitalized for SARS-CoV-2 infection, in the absence of pre-existing autoimmune diseases, might impact their disease severity, affecting overall survival and severe respiratory complication onset.

To gain a better understanding of the association between ANAs and COVID-19, we subsequently evaluated the inflammatory and malnutrition factors associated with COVID-19 pathology. In this regard, a composite measure known as the HALP score, which incorporates hemoglobin, albumin, lymphocyte, and platelet values, has been suggested as a possible indicator for assessing systemic inflammation and malnutrition [14].

The HALP score has been extensively studied to predict the prognosis of cancer patients. Considering the significance of inflammation and malnutrition status in COVID-19 prognosis, it would be advantageous to explore the potential predictive role of the HALP score in COVID-19 patient outcomes [15], thus in association with ANA status.

Antinuclear antibodies (ANAs) and the Hemoglobin-Albumin-Lymphocyte-Platelet (HALP) score play pivotal roles in predicting outcomes in infectious diseases, including COVID-19 [16]. ANAs, traditionally associated with autoimmune disorders, have emerged as potential indicators of disease severity and prognosis in infectious contexts. In COVID-19, ANA presence has been linked to a heightened inflammatory response and an increased risk of severe complications [3]. Conversely, the HALP score, calculated from hemoglobin, albumin, lymphocyte, and platelet counts, serves as a composite prognostic marker associated with mortality and morbidity prediction in infectious diseases [17]. In COVID-19 patients, the HALP score has demonstrated its utility as a predictor of adverse outcomes, showcasing its potential in risk stratification and treatment decision making [18]. Both ANAs and the HALP score stand as valuable tools in the assessment and prognosis of infectious diseases, offering insights into patient outcomes and guiding clinical management strategies.

## 2. Materials and Methods

### 2.1. Study Design and Participants

Among all of the patients hospitalized for COVID-19 at both the Respiratory Diseases Unit and Infectious Diseases Unit at University Hospital “Policlinico Riuniti” of Foggia (Italy) between April 2020 and March 2021, this retrospective, single-center, observational cohort study included 638 incident patients who met the inclusion criteria reported below and signed an informed consent to participate to the present study.

**Figure 1:**
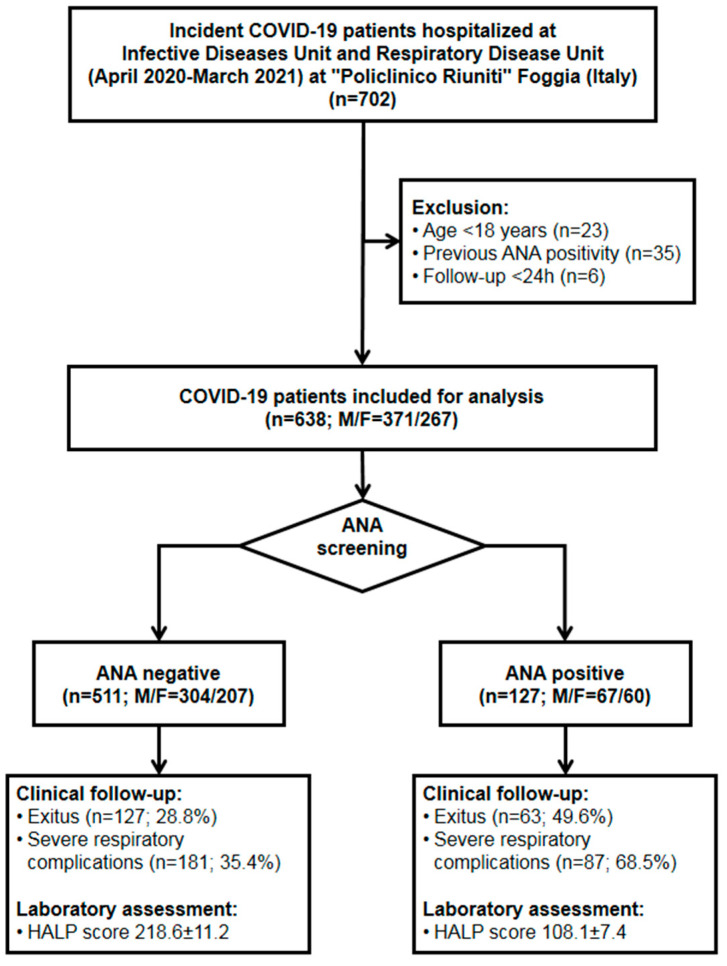
Study design flow chart.

In all of the patients, COVID-19 needing hospitalization was confirmed by both SARS-CoV-2 reverse transcription polymerase chain reaction (RT-PCR) test and by radiological findings of COVID-19-associated pneumonia at X-ray and/or CT examination, as described elsewhere [19]. COVID-19 patients under 18 years of age, as well as those with pre-existing autoimmune diseases or with previously known ANA positivity, were excluded. Furthermore, COVID-19 patients with severe health conditions at hospital admission and a follow-up of less than 24 h were excluded from the study, as shown in Figure 1.

Upon hospital admission, all patients underwent clinical examination and their history of major comorbidities (hypertension, obesity, diabetes, coronary artery diseases, underlying pulmonary diseases, malignancies) was recorded. Laboratory tests included complete blood count [white blood cell count (WBC), lymphocytes, monocytes, neutrophils and thrombocyte count, hemoglobin level], high-sensitivity C-reactive protein (hs-CRP, mg/dL), D-dimer (μg/L), ferritin (ng/mL), fibrinogen (g/L), procalcitonin (μg/L), international normalized ratio (INR), activated partial thromboplastin time (aPTT, sec), and prothrombin time (PT, sec), the results of which were included in the analysis. Moreover, at admission, all patients underwent serum sampling for ANA screening. The clinical and laboratory data, as well as the main clinical outcomes during hospitalization, were recorded in an electronic medical record and then analyzed. The median follow-up during hospitalization was 19 days (range 1–95).

The two primary outcomes of the study were 30-day patient survival and 30-day incidence of severe respiratory complications. COVID-19 patients with severe respiratory complications were defined as subjects with impaired lung function who required mechanical ventilation or who developed shock and/or other organ failure requiring treatment monitoring in the intensive care unit (ICU). The study protocol conformed to the ethical guidelines of the Declaration of Helsinki and was approved by the local ethics committee (decision no. 145/CE/2020 on 30 November 2020; Ethical Committee at University Hospital “Policlinico Riuniti” of Foggia). This was in accordance with the guidelines established by the Regional Ethics Committee for human experimentation.

### 2.2. Lung Function Assessment

Impaired lung function was diagnosed if the patient suffered shortness of breath (≥30 breaths/min), oxygen saturation ≤ 93% at rest, arterial partial pressure of oxygen (PaO_2_)/fraction of inspired oxygen (FiO_2_) ≤ 300 mmHg, or chest imaging showing clear lesion progression within 24–48 h of more than 50%.

### 2.3. Laboratory Assessment

The main laboratory tests, which included complete blood count, clotting test, assessment of renal function, measurement of inflammatory and sepsis markers, and analysis of serum proteins and albumin, were performed at the Central Medical Laboratory of University Hospital “Policlinico Riuniti” of Foggia (Italy), following current laboratory methods, as described elsewhere [20]. 

ANA screening was assessed using the indirect immunofluorescence (IIF) method with HEp-2 cells. In detail, the AESKU ANA-IFA reagent kit and the fully automated HELIOS system were used (Aesku Diagnostics GmbH & Co. KG, Wendelsheim, Germany). Serum samples collected from patients were diluted 1:80 and analyzed according to the manufacturer’s recommendations. The evaluation was performed independently by two clinical pathologists with 10 years of experience using a fluorescence microscope system (Helios system, Aesku Diagnostics GmbH & Co. KG, Wendelsheim, Germany). Positive samples were re-tested and serial dilution (1:160, 1:320, 1:640, 1:1280) was conducted for analysis. In detail, ANA patterns were evaluated according to International Consensus on ANA Patterns (ICAP) standards [21].

### 2.4. HALP (Hemoglobin-Albumin-Lymphocyte-Platelet) Score

An emerging composite index, the HALP (Hemoglobin-Albumin-Lymphocyte-Platelet) score, was evaluated. The HALP score has demonstrated a robust correlation with mortality and morbidity in various inflammatory or proliferative diseases [22]. The HALP score was calculated at hospital admission, according to the following formula: hemoglobin level (g/L) × albumin level (g/L) × lymphocyte count (/L) / platelet count (/L) [23].

### 2.5. Statistical Analysis

Statistical analysis was performed using Statistical Package for Social Sciences (SPSS) 25.0 software (SPSS Inc., Evanston, IL, USA), as previously described [24,25,26,27]. The Kolmogorov–Smirnov test was used to assess variable distribution. The student’s *t*-test for unpaired data or Mann–Whitney U-test was used to compare groups of variables, while the X^2^-test was used to compare groups of frequencies, as appropriate. Correlation between two variables was ascertained by Pearson’s or Spearman’s correlation tests, as appropriate.

Receiver operating characteristic (ROC) curve analysis was performed to validate the association of the HALP score with patient survival and onset of severe respiratory complications.

Subsequently, all of the patients were stratified according to HALP tertiles, and Kaplan–Meier estimates were used to generate both an overall survival curve and a cumulative incidence of severe respiratory complications for COVID-19 patients, while differences among the three groups were assessed by log-rank test. The data were censored if a patient was discharged during the study period. To test the independent effects of different variables on both patient survival and onset of severe respiratory complications, univariate and multivariate Cox regression models were built, and partial correlation coefficients were computed and presented as the hazard ratio and 95% confidence interval (HR; 95% CI). The covariates included in the Cox model were gender, high-sensitivity C-reactive protein (hs-CRP), age, ANA status, and HALP score. Among variables, gender and ANA status were entered as dichotomic values, age was entered as 10-year intervals (<30, 30–40, 40–50, 50–60, 60–70, 70–80, >80 years), hs-CRP as a continuous variable, and the HALP score as tertiles. These variables were included in the multivariate analyses if they had a *p*-value < 0.05 in the univariate analysis or if they were clinically relevant confounders.

All of the data were reported as mean ± SD or as percentage frequency, unless otherwise specified. A *p*-value < 0.05 was considered statistically significant.

## 3. Results

**Figure 2:**
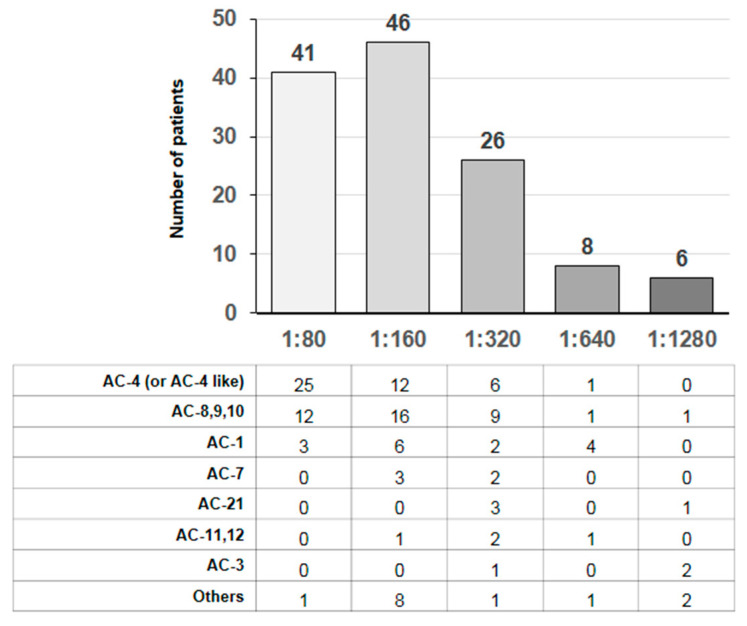
ANA patterns and titers in COVID-19 patients with positive ANA screening. The **upper** histogram shows the number of cases (COVID-19 patients with positive ANA screening) distributed by increasing titer. The **lower** table shows the frequency of the most common ANA patterns in relation to ANA titer. ANA patterns according to International Consensus on ANA Patterns (ICAP) standards (15): AC-1, nuclear homogeneous; AC-3, centromere; AC-4 (or AC-4 like), nuclear fine speckled; AC-7, few nuclear dots; AC-8,9,10, nucleolar; AC-11,12, nuclear envelope; AC-21, mitochondria-like.

Among 638 COVID-19 patients enrolled in this study (M = 371; F = 267), 127 tested positive at ANA screening (19.9%). The algorithm of the study is reported in Figure 1. Briefly, we included a total of 638 patients. Following the ANA antibody screening test, they were divided into two distinct groups: 127 patients tested positive, with a mortality rate of 49.6% and a respiratory complication rate of 68.5%, while 511 patients tested negative for ANA antibodies, with a mortality rate of 28.8% and a respiratory complication rate of 35.4%. It is also noteworthy that 64 patients were excluded due to being under 18 years of age, having previously tested positive for ANAs, and having a follow-up period of less than 24 h. The distributions of both ANA patterns and titers are reported in Figure 2. In detail, most patients exhibited a finely speckled nuclear pattern AC-4 and a titer of 1:160. The main clinical and laboratory features of all patients after stratification into two groups according to ANA status are shown in Table 1. Of note, ANA-positive patients were significantly older than ANA-negative subjects (70.3 ± 18.6 vs. 64.3 ± 17.7 years, *p* < 0.001). Moreover, ANA-positive patients showed significantly lower levels of hemoglobin and albumin, a lower lymphocyte count, and a higher platelet count, as compared to the ANA-negative group (12.7 ± 2.1 vs. 11.7 ± 2.3 g/dL, *p* = 0.015 for hemoglobin; 34.3 ± 7.3 vs. 30.2 ± 7.4 g/L, *p* = 0.028 for albumin; 18.1 ± 11.9 vs. 12.5 ± 11.4%, *p* = 0.043 for lymphocytes; 240 ± 99 vs. 260 ± 112 × 10^3^/mcL, *p* = 0.025 for thrombocytes, respectively).

No significant difference in the other examined laboratory parameters nor in the prevalence of other comorbidities (hypertension, diabetes, coronary artery diseases, underlying lung diseases, malignancies) were observed between the two groups of patients (Table 1).

**Figure 3:**
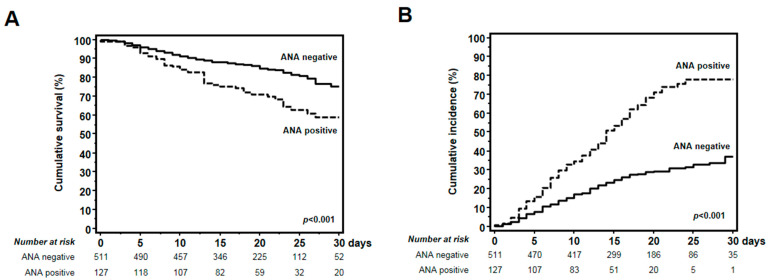
ANA patterns and titers in COVID-19 patients with positive ANA screening. (**A**) The 30-day survival rate of COVID-19 patients with positive ANA screening was significantly lower compared to those with negative ANA screening (64.6% vs. 83.0%, Kaplan–Meier analysis and log-rank test *p* < 0.001). (**B**) The 30-day cumulative incidence of severe respiratory complications increased significantly in COVID-19 patients with positive ANA screening compared to those with negative ANA screening (17.0% vs. 354%, Kaplan–Meier analysis and log-rank test *p* < 0.001).

The overall survival rate in the entire study group was 29.8%. However, lifetime analysis performed after stratification of patients into two groups according to ANA status yielded significant differences. Indeed, COVID-19 patients with positive ANA screening showed a significantly lower 30-day survival rate, as compared to those with negative ANA screening (64.6% vs. 83.0%, Kaplan–Meier analysis and log-rank test *p* < 0.001) (Figure 3A).

Thorough the follow-up, a 42.0% overall cumulative incidence of severe respiratory complications was observed in the entire cohort. Of note, COVID-19 patients with positive ANA screening showed a significantly higher 30-day cumulative incidence of severe respiratory complications, as compared to those with negative ANA screening (35.4% vs. 17.0%, Kaplan–Meier analysis and log-rank test *p* < 0.001) (Figure 3B).

Thus, to explain the different mortality and morbidity rates among COVID-19 patients based on ANA status, we reviewed the entire dataset according to the main laboratory markers of inflammation and malnutrition, which were available in the electronic medical records. Of note, IL-6, a central mediator of COVID-19-related cytokine release syndrome (CRS) toxicity, was not determined in most of the enrolled patients. For this reason, we calculated an emerging composite index, the HALP score, which has shown a strong correlation with mortality and morbidity in other inflammatory or proliferative diseases [22].

**Figure 4:**
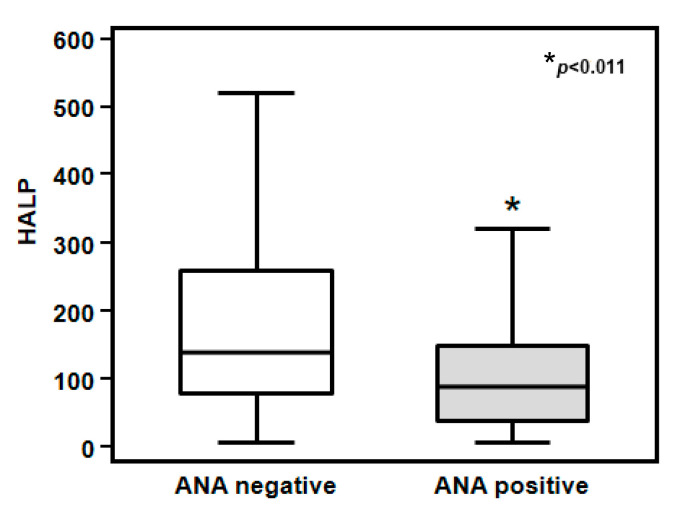
HALP score according to ANA status in COVID-19 patients. The HALP score was significantly lower in COVID-19 patients with positive ANA screening compared to those with negative ANA screening (108.1 ± 7.4 vs. 218.6 ± 11.2 108.1 ± 7.4 AU; *p* < 0.011).

The HALP score was significantly higher in COVID-19 patients with negative ANA screening, as compared to those with positive ANA screening (218.6 ± 11.2 vs. 108.1 ± 7.4 AU; *p* < 0.011) (Figure 4).

**Figure 5:**
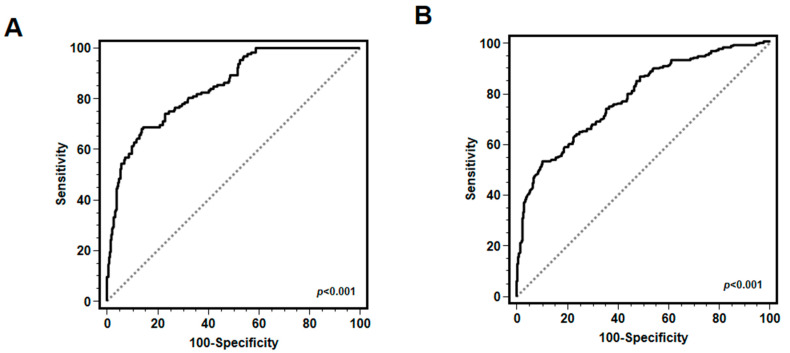
ROC curves for HALP score and 30-day survival and onset of severe respiratory complications in COVID-19 patients. ROC curve analysis identified the HALP score as a significant risk factor for mortality (**A**) and morbidity (**B**) in hospitalized patients with COVID-19 [AUC = 0.845, 95% CI 0.809–0.880, *p* < 0.001 (**A**); AUC = 0.779; 95% CI 0.745–0.811; *p* < 0.001 (**B**)].

ROC curve analysis was carried out to estimate the possible role of the HALP score as a predictor of mortality and morbidity in hospitalized COVID-19 patients. The analysis showed that the HALP score was significantly associated with both 30-day mortality (AUC = 0.845; 95% CI 0.809–0.880; *p* < 0.001) (Figure 5A) and 30-day onset of severe respiratory complications (AUC = 0.779; 95% CI 0.745–0.811; *p* < 0.001) (Figure 5B) in hospitalized COVID-19 patients. 

**Figure 6:**
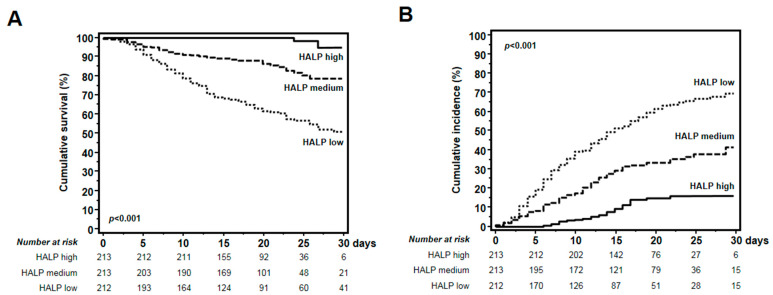
Kaplan–Meier estimates of 30-day survival and cumulative incidence of respiratory complications in COVID-19 patients according to HALP score at hospital admission. All patients were divided into three groups in relation to HALP score tertiles (low HALP ≤ 89.4; middle HALP = 89.4–191.7: high HALP ≥ 191.7). (**A**) COVID-19 patients with low HALP had a 30-day survival rate that was lower than those with high and medium HALP (55.2% vs. 83.6% vs. 99.1% for low, medium, and high HALP, respectively; *p* < 0.001). (**B**) The cumulative incidence of adverse events in COVID-19 patients with low HALP was significantly higher than for those with medium and high HALP (64.6% vs. 35.2% vs. 13.1% for low, medium, and high HALP, respectively; *p* < 0.001).

Thus, lifetime analysis performed after the assignment of all patients to three groups in relation to HALP score tertiles (low HALP ≤ 89.4; middle HALP = 89.4–191.7; high HALP ≥ 191.7) revealed significant differences in the 30-day survival rate for the three groups (*p* < 0.001) (Figure 6A), as well as in the 30-day onset of severe respiratory complications (*p* < 0.001) (Figure 6B).

In detail, an extremely high 30-day survival rate in COVID-19 patients with both high and middle HALP contrasted with that observed in COVID-19 patients with low HALP (99.1% vs. 83.6% vs. 55.2% for high, medium, and low HALP, respectively; *p* < 0.001) (Figure 6A). Conversely, if the lifetime analysis was repeated for the 30-day onset of severe respiratory complications, COVID-19 patients with high and middle HALP showed significantly lower cumulative incidence of adverse events requiring advanced respiratory support (13.1% vs. 35.2% vs. 64.6% for high, medium, and low HALP, respectively; *p* < 0.001).

To estimate the relative risk for both 30-day survival and onset of severe respiratory complications, Cox regression analysis was performed using patient gender and age, C-reactive protein serum levels, ANA status, and the HALP score as covariates (Table 2). Univariate analysis showed that only patient age, ANA status, and the HALP score significantly affected 30-day patient survival (HR 2.099, 95% CI 1.808–2.436, *p* < 0.001 for age; HR 2.066, 95% CI 1.440–2.963 *p* < 0.001 for ANAs; HR 0.249, 95% CI 0.183–0.339, *p* < 0.001 for HALP) (Table 2A). 

The results of the multivariate analysis confirmed a significant effect on patient survival among all of the above covariates (HR 1.789, 95% CI 1.527–2.095, *p* < 0.001 for age; HR 1.802, 95% CI 1.489–2.157, *p* < 0.001 for ANAs; HR 0.363, 95% CI 0.263–0.500, *p* < 0.001 for HALP) (Table 2B). If the Cox regression model was performed for 30-day onset of severe respiratory complications, patient age, ANA status, and the HALP score significantly affected comorbidity onset both in univariate (HR 1.846, 95% CI 1.672–2.038, *p* < 0.001 for age; HR 2.996, 95% CI 2.297–3.910, *p* < 0.001 for ANAs; HR 0.404, 95% CI 0.338–0.483, *p* < 0.001 for HALP) and in multivariate analysis (HR 1.628, 95% CI 1.467–1.807, *p* < 0.001 for age; HR 1.905, 95% CI 1.441–2.518, *p* < 0.001 for ANAs; HR 0.600, 95% CI 0.495–0.726, *p* < 0.001 for HALP). 

Then, we explored a potential association between the HALP score and patient demographic factors. Of note, among the various examined covariates, no correlation was observed with HALP score. Age appeared to be inversely related to the HALP score, although it did not reach statistical significance (*p* = 0.091). Moreover, no differences in median HALP score were observed if patients were stratified for gender (*p* = 0.276). Finally, no correlation was observed between the HALP score and ANA titers (*p* = 0.187).

## 4. Discussion

In this retrospective, observational clinical study, we explored the clinical significance of ANA positivity in COVID-19 patients by examining the association between COVID-19-related clinical outcomes and autoantibody onset. We found that ANA-positive patients developed more severe disease, with a worse prognosis and increased mortality and morbidity, as compared with ANA-negative patients.

The potential relationship between COVID-19 and autoimmune phenomena has been widely analyzed in many reports. The onset of ANAs is usually associated with the development of autoimmune diseases, and there is evidence that SARS-CoV-2 infection may be correlated with the onset autoimmune phenomena [28]. Several studies have reported the presence of antinuclear antibodies (ANAs) in more than 35% of COVID-19 patients. Additionally, several reports indicate the prevalence of anti-Ro/SSA in 25%, rheumatoid factor in 19%, lupus anticoagulant in 11%, and antibodies against interferon (IFN)-I in 10% of COVID-19 patients [7,10,29]. A possible suggested mechanism may be the immune mimicry between 28 human proteins with homologous areas to SARS-CoV-2 peptides [30]. Alternative proposed mechanisms involve bystander activation triggered by a hyper-inflammatory condition, commonly referred to as a “cytokine storm” or “cytokine release syndrome”, viral persistence leading to polyclonal activation due to continuous exposure to viral antigens controlling immune-mediated injury, and the formation of neutrophil extracellular traps [31,32]. Within individuals affected by COVID-19, the range of autoimmune-related symptoms spans from organ-specific to systemic autoimmune and inflammatory disorders [33,34]. However, in our cohort, we did not observe autoimmune symptoms but only ANA positivity in almost 20% of COVID-19 patients at hospitalization without previous diagnosis of autoimmune diseases. In particular, ANA positivity seemed to be associated with a poor prognosis in these patients.

In accordance with our findings, previous studies showed that the clinical course of SARS-CoV-2 infection was strongly affected by ANA status [6,35]. A previous report showed that patients with COVID-19 with a higher rate of ANA positivity had a worse clinical course [12]. Another study showed that COVID-19 patients with a poor prognosis exhibited a higher percentage of autoantibodies, thus being good predictors of worse outcome [36].

To date, no study has deeply investigated the possible relationship between SARS-CoV-2 infection and ANA development. ANAs, usually used as markers of autoimmune diseases, may occur after transient reactivation of autoreactive B and plasma cells following infection [37].

In the context of severe COVID-19, the viral-induced upregulation of extrafollicular B cells, including clonotypes that are autoreactive, may produce autoantibody-secreting cells [38].

Taken together, the available data suggest that the onset of ANAs may not only be an immune epiphenomenon of immune system hyper-activation and dysregulation but also a possible predictive marker of clinical outcome in COVID-19 patients.

Interestingly, most patients with ANA positivity showed nuclear granular or nucleolar patterns with mainly medium-low titers, but no correlation was observed between ANA patterns or titers and major clinical outcomes.

In this scenario, the pro-inflammatory cytokine interleukin-6 (IL-6), widely explored in the setting of COVID-19 pathophysiology, may also play a key role in the onset of de novo ANAs [39,40]. 

IL-6 is a pro-inflammatory cytokine produced by various cells, including macrophages and B and T cells, in response to inflammatory stimuli. This cytokine plays an important role in regulating the immune response. In detail, IL-6 can contribute to stimulate B cell differentiation into plasma cells and promote their antibody production, thus contributing to immunity [41].

Elevated levels of IL-6 have been associated with autoimmune conditions, in which the immune system mistakenly attacks healthy tissues. In these pathologies, IL-6 can contribute to the activation and survival of B lymphocytes producing autoantibodies [42,43]. In detail, IL-6 is able to increase B cell IgG production and its release by B cells themselves may promote T follicular helper cell differentiation, spontaneous germinal center formation, and class-switched autoantibody production during humoral autoimmunity [44,45]. For these reasons, IL-6 also probably plays a crucial role in de novo ANA development in COVID-19 patients.

Unfortunately, IL-6 titration was not available for all our patients as this assay has only been routinely performed in our laboratory since January 2021, thus excluding a large portion of our enrolled patients. For this reason, we focused our analysis on available laboratory markers.

Due to the key role of inflammatory responses to SARS-CoV-2 infection in the development of COVID-19 features, we aimed to analyze the role of inflammation markers in relation to ANA status. C-reactive protein levels were elevated in both ANA-positive and-negative patients and, thus, was unable to predict patient outcome. Then, to provide an explanation of the disparities in mortality and morbidity rates among COVID-19 patients based on ANA status, we analyzed additional inflammation and malnutrition markers in our cohort. Due to the lack of IL-6 values in most of the patients studied, we chose to analyze the HALP score, a composite index of inflammation and malnutrition that is well connected to mortality and morbidity in other diseases. Literature evidence suggests that a low HALP score could be used to predict mortality in cancer patients [14,46,47].

Various studies have examined the prognostic importance of each factor of the HALP score in COVID-19 patients. The reduction in hemoglobin levels in COVID-19 patients at the time of hospitalization was demonstrated to be a predictor of mortality [48,49,50].

Adequate nutrition has a positive impact on the immune response, and a positive correlation has been found between poor nutritional status and a longer stay in intensive care and lymphopenia [51]. Hypoalbuminemia has been observed in patients with nutritional problems and it has been shown to be associated with mortality [52,53]. Recent studies that are consistent with these data suggest that COVID-19 patients with low albumin levels may have a more severe disease course, leading to a higher mortality rate [54,55]. In COVID-19 patients, reduced survival was associated with low levels of lymphocytes and platelets, although the cause was not fully explained [56,57,58,59,60,61]. 

Our study revealed a significant lower HALP score in ANA-positive patients compared to ANA-negative patients, thus suggesting that lower HALP status at COVID-19 diagnosis is associated with high mortality and respiratory complications. Moreover, both ROC analysis and Kaplan–Meier survival analysis confirmed the predictive role of the HALP score for worse 30-day mortality and higher severe respiratory complications in hospitalized COVID-19 patients.

Previous studies have extensively analyzed the role of different inflammatory markers (hs-CRP, IL-6) as well as ANA status as predictors of in-hospital morbidity and mortality in COVID-19 patients at different stages of disease severity, but with variable and not always easily reproducible results [56,62]. Our study, although monocentric, proposes a simple laboratory tool (HALP score), easily reproducible in every hospital, with the aim of predicting major clinical outcomes in hospitalized COVID-19 patients (AUC = 0.845, *p* < 0.001 for 30-day mortality; AUC = 0.779, *p* < 0.001 for severe respiratory complications), even if further confirmatory studies are needed.

Taken together, our data, although limited to a single center, suggest that the HALP score may be a good predictor of major clinical outcomes in COVID-19 patients.

Undoubtedly, this study is limited by being a single-center analysis and by the unavailability of serial monitoring of all hematological and biochemical parameters, including ANA levels. However, we attempted to eliminate certain confounding factors in this study. We acknowledge that, except for a limited number of cases, we lacked information on antinuclear antibody (ANA) serum levels before hospitalization for the majority of the enrolled patients, undoubtedly representing a limitation of the study. However, it is noteworthy that none of the enrolled patients had a history of autoimmune disease, and none of those with ANA positivity subsequently developed an autoimmune disease at the 1-year follow-up after discharge. Moreover, to avoid the confounding effect of vaccine status on major clinical outcomes, we limited our retrospective analysis to COVID-19 patients hospitalized between April 2020 and March 2021 before the mass vaccination campaign in Italy could unfold its beneficial effects.

While the results of our study are completely preliminary, further studies are needed to better explore the link between the onset of autoimmune phenomena and clinical outcomes in diseases characterized by a preponderant inflammatory and auto-inflammatory component such as COVID-19.

## 5. Conclusions

Identifying different markers to better understand the etiopathogenesis and evolution of COVID-19 has been made possible by the scientific community’s efforts. Our research indicates that ANA levels are increased in severe COVID-19 cases, and it seems that their presence is related to a more aggressive disease phenotype with a low survival rate. In ANA-positive patients with COVID-19, a low HALP score had a significant correlation with mortality and morbidity, as reported. Our findings strongly suggest that the HALP score is a valuable indicator of mortality in COVID-19 patients. Despite the HALP score being primarily studied in patients with cancer or other inflammatory diseases, future research will likely investigate the combination of the HALP score with other scores to obtain more specific information about the prognosis of different diseases.

Although this study offers promising results, it has limitations that could affect its global applicability given the single-center nature and the specific analysis period used.

Furthermore, the absence of serial monitoring of autoimmune markers is a factor to consider. Further large-scale multicenter studies are crucial to confirm our findings in different clinical settings.

## Figures and Tables

**Table 1:** Baseline clinical and laboratory characteristics of COVID-19 patients with negative and positive ANA screening at admission to the hospital.

	Total (*n* = 638)	ANA Negative (*n* = 511)	ANA Positive (*n* = 127)	*p*-Value
***Clinical characteristics***				
Female gender, *n* (%)	41.8%	40.5%	47.2%	0.191
Age (years)	65.5 ± 18.0	64.3 ± 17.7	70.3 ± 18.6	<0.001
Hospital stay (days)	22 ± 15	22.1 ± 15.1	22.3 ± 16.5	0.876
Comorbidities				
Hypertension, *n* (%)	227 (35.9%)	178 (34.8%)	51 (40.5%)	0.310
Diabetes, n (%)	123 (19.3%)	97 (19.0%)	26 (20.5%)	0.798
Obesity (BMI > 25), n (%)	108 (16.9%)	84 (16.4%)	24 (18.9%)	0.508
CAD, n (%)	48 (7.5%)	35 (6.8%)	13 (10.2%)	0.195
Underlying PD, n (%)	34 (5.3%)	24 (4.7%)	10 (7.9%)	0.154
Malignancies, n (%)	31 (4.9%)	22 (4.3%)	9 (7.1%)	0.192
***Laboratory Assessment***				
hs-CRP, mg/L	68.5 ± 66.1	68.2 ± 72.7	69.4 ± 67.7	0.869
Procalcitonin, mcg/L	0.21 ± 0.26	0.18 ± 0.20	0.25 ± 0,21	0.296
Fibrinogen, g/L	5,43 ± 1.86	5.51 ± 2.14	5.11 ± 0,99	0.732
D-dimers, ng/mL	3140 ± 376	3193 ± 325	2927 ± 1310	0.859
PT, sec	13.3 ± 6.8	13.4 ± 7.4	12.9 ± 3.6	0.343
aPTT, sec	32.0 ± 5.2	32.3 ± 5.2	30.8 ± 4.9	0.108
INR, ratio	1.18 ± 0.53	1.18 ± 0.58	1.17 ± 0.91	0.687
Ferritin, mcg/mL	435 ± 483	452 ± 508	369 ± 428	0.476
WBC, ×10^3^/mcL	8.73 ± 4.58	8.62 ± 4.73	9.17 ± 3.89	0.169
Lymphocytes, %	16.9 ± 11.8	18.1 ± 11.9	12.5 ± 11.4	0.043
Monocytes, %	5.1 ± 2.0	4.9 ± 2.1	5.7 ± 1.9	0.637
Neutrophils, %	76.3 ± 14.6	76.1 ± 14.4	77.1 ± 14.8	0.662
Thrombocytes, ×10^3^/mcL	244 ± 102	240 ± 99	260 ± 112	0.025
Hemoglobin, g/dL	12.5 ± 2.1	12.7 ± 2.1	11.7 ± 2.3	0.015
Creatinine, mg/dL	1.58 ± 0.41	1.66 ± 1.51	1.23 ± 1.61	0.458
Protein, g/L	65.5 ± 7.8	65.7 ± 7.7	64.5 ± 8.5	0.147
Albumin, g/L	33.5 ± 7.4	34.3 ± 7.3	30.2 ± 7.4	0.028

Values are expressed as mean ± SD or number of cases and (percentage) for frequencies. A comparison between the main clinical and laboratory parameters of COVID-19 patients with positive vs. negative ANA screening is shown and *p*-values are reported. Significant *p*-values are in bold. Abbreviation: a-PTT, activated partial thromboplastin time; BMI, body mass index; CAD, Coronary Artery Diseases; hs-CRP, high sensitivity C-reactive protein; INR, international normalized ratio; PD, pulmonary diseases; PT: prothrombin time; WBC, white blood cell count.

**Table 2:** Univariate and multivariate regression analysis of factors affecting 30-day survival (**A**) and incidence of severe respiratory complications (**B**) in COVID-19 patients.

A	Univariate Analysis 95% CI				Multivariate Analysis 95% CI			
	HR	Lower	Higher	*p*-Value	HR	Lower	Higher	*p*-Value
Gender ^1^	1.143	0.811	1.610	0.445	-	-	-	-
hs-CRP ^2^	1.020	0.985	1.057	0.256	-	-	-	-
Age ^3^	2.099	1.808	2.436	<0.001	1.789	1.527	2.095	<0.001
ANA status ^1^	2.066	1.440	2.963	<0.001	1.802	1.489	2.157	<0.001
HALP score ^4^	0.249	0.183	0.339	<0.001	0.363	0.263	0.500	<0.001
**B**	**Univariate Analysis** **95% CI**				**Multivariate Analysis** **95% CI**			
	**HR**	**Lower**	**Higher**	***p*-Value**	**HR**	**Lower**	**Higher**	***p*-Value**
Gender ^1^	1.222	0.948	1.575	0.122	-	-	-	-
hs-CRP ^2^	1.003	0.966	1.041	0.883	-	-	-	-
Age ^3^	1.846	1.672	2.038	<0.001	1.628	1.467	1.807	<0.001
ANA status ^1^	2.996	2.297	3.910	<0.001	1.905	1.441	2.518	<0.001
HALP score ^4^	0.404	0.338	0.483	<0.001	0.600	0.495	0.726	<0.001

^1^ Gender and ANA status were entered as dichotomic values; ^2^ High sensitivity C-reactive protein (hs-CRP) was entered as a continuous variable; ^3^ Age was entered as 10-year intervals (<30, 30–40, 40–50, 50–60, 60–70, 70–80, >80 years); ^4^ HALP score was entered as tertiles.

## Data Availability

The datasets used and/or analyzed during the current study are available from the corresponding author upon reasonable request.
